# Eyes and ears: A comparative approach linking the chemical composition of cod otoliths and eye lenses

**DOI:** 10.1111/jfb.15159

**Published:** 2022-07-29

**Authors:** Jonathan Stounberg, Tonny Bernt Thomsen, Benjamin Dominguez Heredia, Karin Hüssy

**Affiliations:** ^1^ National Institute of Aquatic Resources Technical University of Denmark Lyngby Denmark; ^2^ Geological Survey of Denmark and Greenland Copenhagen Denmark

**Keywords:** Atlantic cod, chemical profile, fish eye lenses, growth rings, LA‐ICPMS, otoliths

## Abstract

Fish eye lenses are a protein‐based chronological recorder of microchemical constituents that are a potentially useful tool for interpretations of environmental, ecological and life‐history experienced by fish. Here, we present the first study with data on the chemical composition of eye lenses from Baltic cod examined using laser ablation inductively coupled plasma mass spectrometry (LA‐ICPMS) and compare these spatially resolved data to otoliths from the same fish also analysed by LA‐ICPMS, measuring the isotopes ^27^Al, ^137^Ba, ^43^Ca, ^52^Cr, ^65^Cu, ^57^Fe, ^39^K, ^7^Li, ^25^Mg, ^55^Mn, ^31^P, ^208^Pb, ^85^Rb, ^45^Sc, ^29^Si, ^88^Sr, ^47^Ti, ^50^V, ^149^Yb, ^66^Zn and ^90^Zr. Comparison of the variation in element concentrations between eye lenses and otoliths from the same individuals showed minor similarities, suggesting a different governance in the uptake processes. A strong overlap between the concentric growth rings in the eye lenses and the otolith Sr periodicity was observed, where each consecutive minima in the chemical profile with high accuracy correspond to the width of each lens ring. No comparable trends were seen between growth rings and all other elements measured from both lenses and otoliths. The characteristic rings observed in cod eye lenses do not seem to represent seasonal fluctuation nor are they found to be directly linked to age. With this research, we provide a baseline study identifying elements in corresponding eye lenses and otoliths that show potential for unravelling the environmental and biological conditions experienced by fish.

## 1 INTRODUCTION

Otoliths are a useful tool in fisheries ecology because they grow incrementally throughout the fish's life. These growth increments will in temperate areas form visible seasonal rings that represent years. Moreover, the otolith is not subject to resorption (Campana, 1999; Campana & Thorrold, 2001). Although the most widely used structure for fish aging, otoliths are not the only tissue that exhibit such traits. The endoskeleton, fin spines, fin rays, scales and eye lenses have similar features (Pourang *et al*., 2018; Tzadik *et al*., 2017). Of these, the scales are the most widely used in age estimation, as sampling of these is easy and nonlethal. Otolith chemical composition is also extensively used to study migration patterns and connectivity, as well as population and subpopulation structures. The chemical composition of otoliths is regulated by a suite of extrinsic (*e*.*g*., water concentration, temperature) and intrinsic (*e*.*g*., fish size, growth, sex and food) factors (Anon, 2014; Campana *et al*., 1996; Hüssy *et al*., 2020; Sturrock *et al*., 2012, 2015). Patterns within and between individuals yield information on life‐history events such as migration patterns and stock structure (Campana, 2005; Campana *et al*., 1996; Campana & Thorrold, 2001; Svedäng *et al*., 2010), and in recent years even direct age (Heimbrand *et al*., 2020). Other aspects explored with the various chronological tissues have included diets and ecosystem reconstruction (Tzadik *et al*., 2017).

Among the chronological tissues, eye lenses are a particularly interesting candidate for inferring new knowledge about fishes' life history. Unlike otoliths and many other chronological tissues, eye lenses have a protein‐based structure (Pourang *et al*., 2018). They consist of a wide range of proteins bound in primarily α and γ crystallines that are thought to make up the structural part of the eye lens (Dove, 1999; Wistow & Slingsby, 2016). The exact construction of these crystallines is less well documented, although it is known that a main component is sulphur (Mahler *et al*., 2013). Studies on the microchemistry of fish eye lenses are limited and tend to focus on a few selected elements. Some of these studies (*e*.*g*., Quaeck, 2017; Quaeck‐Davies *et al*., 2018) have examined the structural design of eye lenses from various species and the behaviour of the physical structure of the lens. These studies have established that eye lenses are a good repository for stable isotope‐derived information relating to the entire life history of a fish (Young *et al*., 2022). Other studies (Gillanders, 2001; Kingsford & Gillanders, 2000; Pourang *et al*., 2018) have examined the use of eye lens microchemistry in a fisheries management context. Some of these studies show that eye lens chemistry has potential in stock discrimination using elemental fingerprinting, similar to the use of otoliths (Campana, 2005). Other studies have linked eye lens trace elements with environmental factors such as depth, spatial scale and heavy metal pollution (Dove, 1999; Dove & Kingsford, 1998). No study exists to link trace elements in the fish blood plasma with eye lens concentrations like for otoliths (Sturrock *et al*., 2012, 2013, 2014). This is nevertheless an essential feature to fully understand the uptake processes of trace elements to any chronological tissue and to evaluate to what extent trace elements reflect environmental concentrations or physiological processes.

In this study, the microchemistry of corresponding eye lenses and otoliths from Atlantic cod (*Gadus morhua*) was investigated with the aim of (a) establishing a baseline of trace elements occurring in eye lenses using solution inductively coupled plasma mass spectrometry (ICPMS), (b) providing profiles of eye lens element composition using laser ablation ICPMS (LA‐ICPMS), (c) comparing element concentrations of eye lenses and otoliths and assessing the influence of physiological factors such as size, sex and maturity, and (d) correlating the growth ring structure visible in eye lenses with the chemical profiles of both the eye lenses and the otoliths, aiming to explain what the rings represent.

## 2 MATERIALS AND METHODS

### 2.1 Sample collection

**FIGURE 1:**
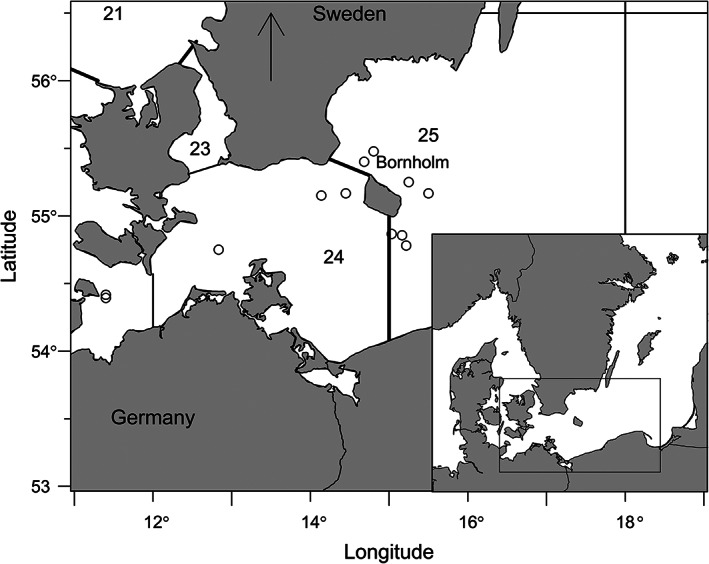
Map of the southern Baltic Sea with sampling locations of the Atlantic cod (*Gadus morhua*) used in this study. Numbers indicate ICES (International Counsil for the Exploration of the Sea) subdivisions: 23 = sound, 24 = western Baltic Sea and 25 = eastern Baltic Sea. The solid lines 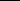 indicate the border between ICES subdivisions

Otoliths and eye lenses of 12 Baltic cod were collected randomly from fisheries samples between 2017 and 2019. Following capture, the length, weight, sex, maturity stage and capture location were recorded. Eye lenses and otoliths were dissected out, dried and stored in paper envelopes. An overview of the biological data and the capture positions of the sampled cod are shown in Table 1 and Figure 1, respectively. Genetic stock identification was used to ensure that all cod belonged to the eastern Baltic cod population (for details see Hansen‐Hemmer *et al*., 2018). For nine cod, sex and maturity stage were available, whereas only size and capture position were available for the remaining three individuals. Age estimation of eastern Baltic cod is highly uncertain due to a combination of environmental and biological factors (see the review by Hüssy *et al*., 2016), therefore no estimate of age is available for these samples.

**TABLE 1:** *Gadus morhua* (cod) sampled, with size, sex, maturity stage, and capture year and month, for which the chemical profile for both eye lenses and otoliths are available

Fish ID	Size (mm)	Sex	Maturity stage	Year	Month
1	432.52	M	2	2017	6
2	355.00	F	3	2017	5
3	486.58	F	4	2017	9
4	401.66	NA	NA	2017	5
5	470.26	F	5	2017	7
6	398.86	M	2	2019	1
7	412.88	NA	NA	2017	6
8	376.42	M	2	2019	1
9	400.90	F	2	2019	1
10	373.10	NA	NA	2019	1
11	430.48	F	2	2019	2
12	453.94	F	4	2018	10

### 2.2 Chemical analysis

The sampled otoliths were cleaned using an ultrasonic bath with deionized water for 10 min, rinsed with deionized water and left to dry in acid‐washed trays in a laminar flow hood following the processing protocol by Hüssy *et al*. (2021). Lenses were cleaned by removing any adhering tissue and dried for at least 12 months. Lenses and otoliths were then embedded in Struers (Detroit Rd. Westlake, cleavland, OH, USA) cold mounting casting epoxy and sectioned through the core with an Accutom‐100 multicutter to expose the entire growth axis from core to edge. The surface of each section was polished using 3 μm abrasive paper mounted on Buehler (Waukegan Rd, Lake Bluff, IL, USA) rotating disks, and then cleaned once more as described above. The polished otolith and lens sections were digitized using a Leica (Wetzlar, Germany) DCF290 camera with a magnification of 380 μm pixel‐1 in a standard setup with 8 bit/channel and 2048 × 1536‐pixel frame. These images were used to place the transect line for the LA‐ICPMS analyses.

**FIGURE 2:**
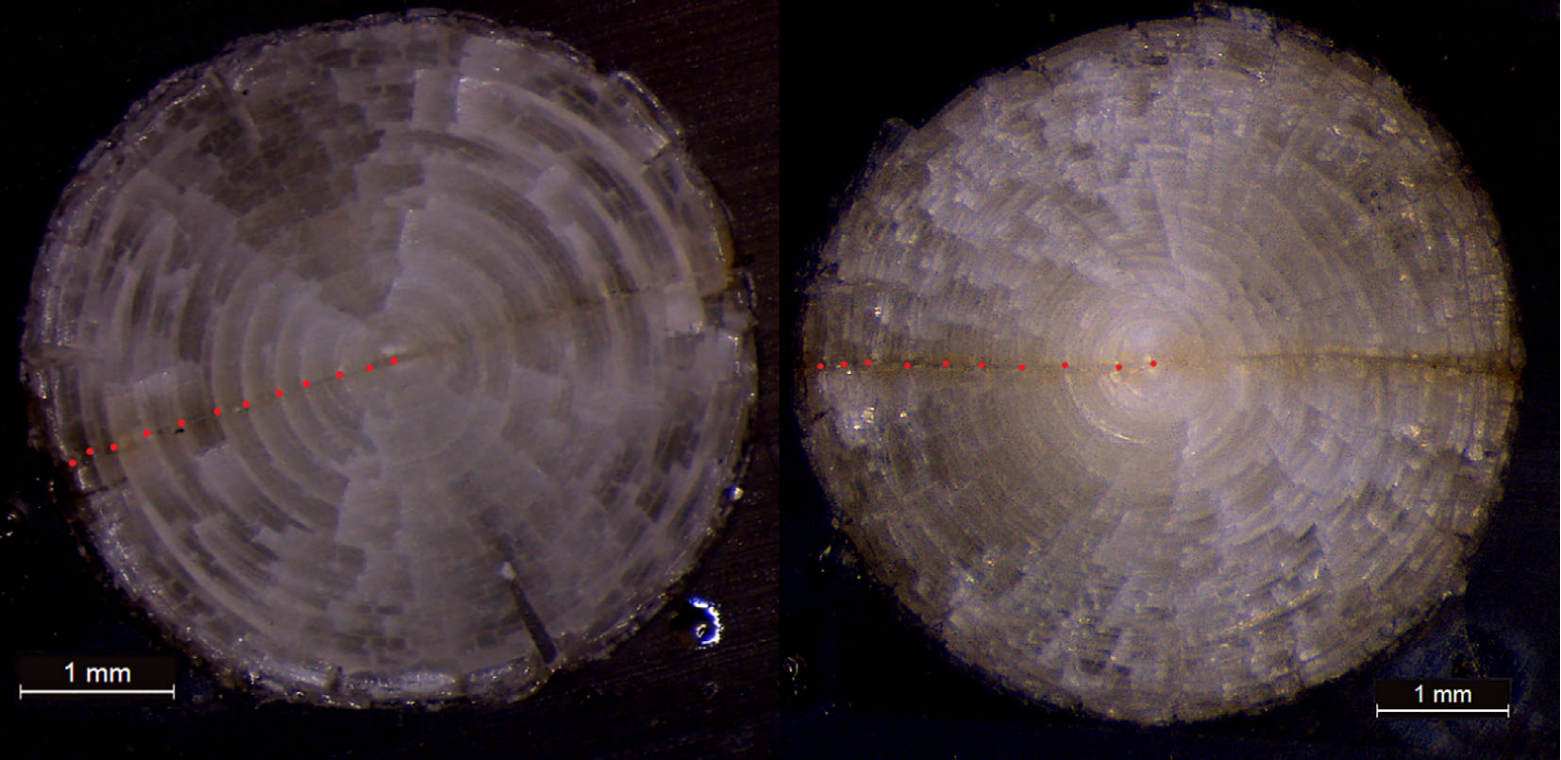
Images of *Gadus morhua* eye lenses of two selected individuals (fish ID #3 and #5), where the red dots 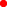 represent the growth rings identified along the laser transect. The left image shows an ideal example with well‐defined growth rings and the right image shows an example where the growth rings are difficult to discern

The microchemical analyses were carried out at the Geological Survey of Denmark and Greenland (GEUS). Following the protocol of Gillanders (2001), solution‐based ICP‐MS was used to identify those elements in the eye lenses that occur in a concentration level suitable for LA‐ICPMS analysis. Eye lenses from three cod were dissolved and used for these analyses. Semiquantitative abundances for the elements were obtained from the solution ICP‐MS analyses, providing averaged measures for the element concentration in the eye lenses that was used as a reference point for quality asurrence/controle (QA/QC) of the LA‐ICPMS analyses. In this study, 22 isotopes (^27^Al, ^137^Ba, ^43^Ca, ^52^Cr, ^65^Cu, ^57^Fe, ^39^K, ^7^Li, ^25^Mg, ^55^Mn, ^61^Ni, ^31^P, ^208^Pb, ^85^Rb, ^45^Sc, ^29^Si, ^88^Sr, ^47^Ti, ^50^V, ^149^Yb, ^66^Zn, ^90^Zr) were included in the LA‐ICPMS analysis. During LA‐ICPMS analyses the variation in the elemental abundances in the lenses and otoliths was measured along a transect line running from core to edge in the lenses (example shown in Figure 2) and from core to the dorsal edge in the otoliths. The LA‐ICPMS run conditions are listed in Supporting Information Table S1.

The basic analytical approach and data processing techniques used for analysing otoliths at GEUS are described in Serre *et al*. (2018). Additional details and LA‐ICPMS settings are included in Supporting Information Table S1, including the analytical precision and accuracy of the LA‐ICPMS data (Supporting Information Figure S1). The LA‐ICPMS analyses are reported in counts per second (cps) for each isotope measured along the transect. To calculate elemental abundances (*e*.*g*., in ppm) we use an internal standard element and an external standard reference matrix (*e*.*g*., NIST‐610 glass) with known element concentrations. For otoliths, Ca was used as the internal standard element due to the otolith's robust calcium carbonate structure, which was assumed to be 38.3 wt.% Ca (Serre *et al*. 2018 and references therein) for all otoliths analysed. The otolith data used here were previously included in Hüssy *et al*. (2021). For eye lenses P was selected as the best internal standard element based on its consistency in apparent concentration as measured by the solution‐based ICP‐MS analyses, and because P in LA‐ICPMS pre‐run test analyses together with Mg showed the smallest variation of the elements measured across the growth zones of the eye lenses. A P averaged abundance of 0.09 wt% P_2_O_5_ (corresponding to *ca*. 390 ppm P) as determined by solution ICP‐MS was assumed to be representative for all the eye lenses analysed. Variations in Ca content across the transects in the otoliths and P in the eye lenses may of course occur, but there was not the scope here to examine all otoliths and eye lenses in detail for variations across the growth zones. In this study we thus assume a more‐or‐less consistent Ca and P content for the otoliths and eye lenses, respectively. Consequently, any significant variation in the abundance of these internal standard elements will affect the accuracy of all other element concentrations, but it does not affect element ratios determined relative to Ca or P for the otoliths and eye lenses, respectively.

### 2.3 Eye lens ring identification

As for otoliths, growth zones in eye lenses are concentrically and symmetrically formed around the core. Unlike in otoliths, there is no consensus as to what these rings represent or why they form in this manner. For each cod we have used the optical images of the cross‐sectioned eye lenses to identify these rings. ImageJ (v. 1.53e) was used to measure the width of each ring along the laser transect line from the core to both the left and right lens edges. Not all individuals showed a clear and easily recognizable outline of the rings. Figure 2 shows an example of (a) easy identifiable rings and (b) rings that were difficult to identify. Where the structure was less clear, *e*.*g*., where rings appear to have ‘merged’ or ‘dissipated’, the complete rings were identified by superimposing a full circle on the image using an elliptical tool in ImageJ. Any ‘incomplete’ rings were not considered true rings.

### 2.4 Data analysis

It was assumed that the chemical profile of each individual is statistically independent, thus outlier removal was performed on each eye lens separately, applying the same procedure for all samples (Zuur *et al*., 2010). Each individual transect represents a timeline and some elements exhibited large shifts in the concentration along the data transect. To account for the shifts that would otherwise offset standard deviation and mean value estimates, the data from each individual were partitioned into four equal parts for each element, respectively, illustrated by the vertical lines in Supporting Information Figure S2. Within each section of the partitioned data, any measurements greater than section mean ± 4*σ* were excluded.

The quality of the laser transect data for both the eye lenses and otoliths was examined by calculating the signal‐to‐noise‐ratios (SNRs), defined as mean^2^/s.d. ^2^. This determined which elements showed overall potential for meaningful statistical modelling (SNR > 5).

A direct comparison of the element concentrations of lenses and otoliths was explored trough statistical modelling. The LA‐ICPMS data were aggregated per individual into three datasets for modelling: (1) the mean of the whole data transects, using ‘all’ data points; (2) the mean of the ‘core’, defined as the average number of measurements from lens core to the first ring (first 20 measurements corresponding to 0.2 mm) (this distance corresponds proportionally in size to 50 measurements in the otolith, since both otoliths and lenses are linearly proportional to fish length); and (c) the mean of the ‘edge’, defined as the average number of measurements from approximately 1 year of the fishes lifespan, found from the T‐bar anchor tags in otoliths, 50 measurements for otoliths and 20 for the lenses. We tested for concentration differences between the eye lenses and the otoliths. In addition, we tried to establish if any lens and otolith concentrations showed the same response to biological factors included here. For elements with SNR > 5, mixed linear models were set up using the R function ‘lmer’ (Kuznetsova *et al*., 2017). For this analysis, the factor *type* was used, denoting either lens or otolith concentration. Fish ID number was added as a random effects term to account for differences in data points between lenses and otoliths. The general full model for the ‘all’ measurements, for each of the elements considered, were constructed as follows:

(1) $$Y_{i}=\mu +a_{1}\left(\mathrm{typ}e_{i}\right)+a_{2}\left(\mathrm{se}x_{i}\right)+\beta_{1}\left(\mathrm{typ}e_{i},\mathrm{se}x_{i}\right)+d\left(\text{fishID}\right)+\varepsilon_{i}$$

where *Y* represents each element modelled, *i = 1*, *…* 24 (two measurements per individual), *d*(*fishID*) ∼ *N*(0,*σ*_*fishID*_^2^) and $\varepsilon_{i}∼N\left(0,\sigma^{2}\right)$. Furthermore, it is assumed that all *d*(*fishID*) and *I* values are independent. Model validation through residuals check, Boxcox *etc*. and necessary data transformations was performed. Model reduction was done using ANOVA tables together with $\chi^{2}$‐tests.

Large physiological changes happen over the course of the fish's life. This may affect how the different elements are taken up by the tissues. To test whether any effects were masked by taking the average over the whole growth axis (representing the entire life span of the fish), data representing the start and end of the life span were analysed with additional mixed linear models, focusing on effects more predominant in these life stages. For ‘core’ and ‘endge’ the model corresponded exactly to model (1). Given different maturity stages among the samples it would have been interesting to include the maturity stage (*mastg*) in the ‘edge’ analysis, but the data were found to be insufficient to support this analysis.

An advantage of LA‐ICPMS analysis is that the full chronological information from hatch to death is recorded, where each data point represents an actual period in time of the fish's life. This approach was used by Heimbrand *et al*. (2020) to look for seasonal patterns in elements of cod otoliths, matching the physical year rings visible to element trends. To test if similar seasonal or periodic variation occur in the elements of cod eye lenses, the R‐function ‘loess’ (Cleveland *et al*., 1992) was used for local second‐degree polynomial regressions for each element and all individuals. Regression with low standard error for all predictions were achieved for the elements Cu, K, Mg, P, Sr and Zn. The R‐function ‘IDPmisc$peaks’ was then used to identify the maxima and minima of the loess predictions, where a minimum peak height detection was set for each element individually, dependent on the scale of the concentration. To test for possible coherence between uptake of a given element to the otolith and the formation of the eye lens rings, the same procedure of local polynomial regression was done for the otolith element transects, first presented in Hüssy *et al*. (2021). Otolith elements Ba, K, Mg, Mn, P and Sr were annotated proportionally to the lens rings. For this annotation, it is assumed that both otoliths and eye lenses grow continuously throughout the fish's life, and both in proportion to the fish growth, so that the axis lengths of otolith and eye lenses are proportional at all increments (Quaeck‐Davies *et al*., 2018).

**FIGURE 3:**
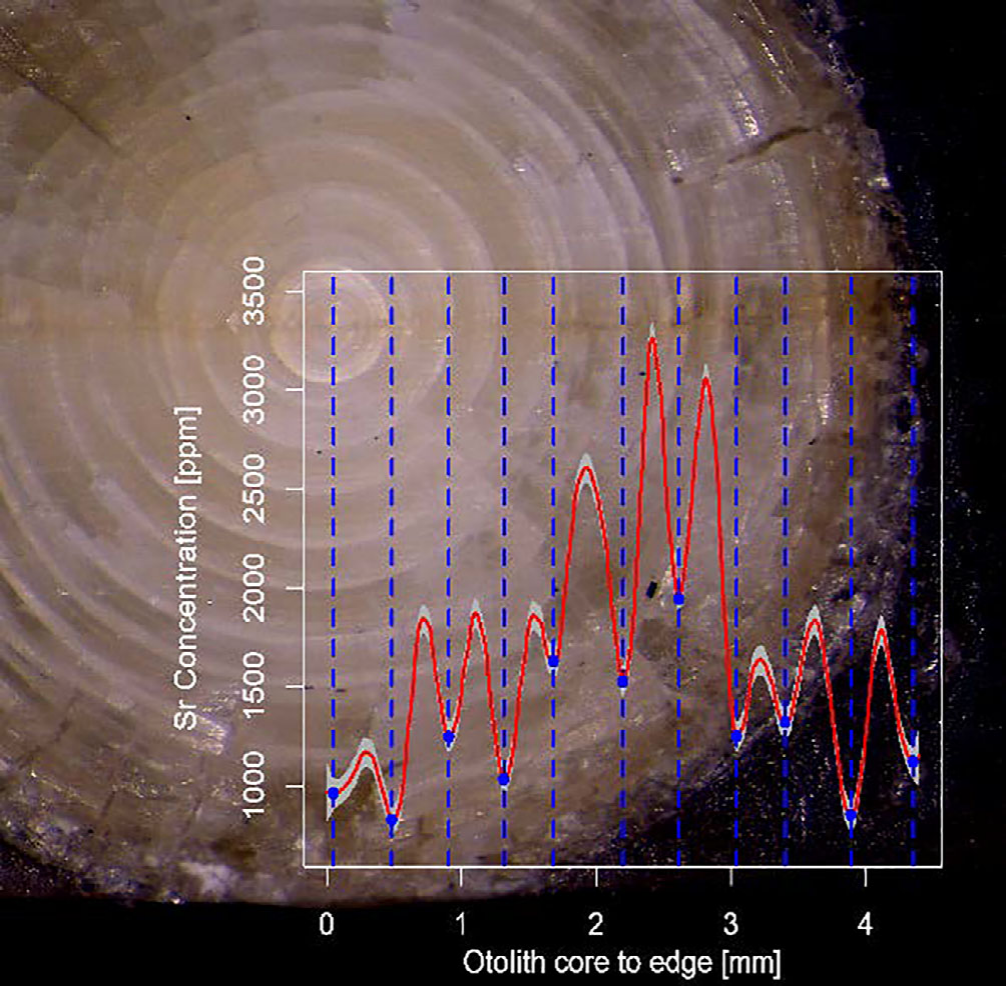
Image of a *Gadus morhua* eye lens (fish ID #3) illustrating the laser transect from the lens core to the edge. Superimposed on the image the chemical profile of otolith Sr for illustrating the correlation in occurrence of otolith Sr minima and eye lens rings. The red line 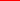 is the fitted local second‐degree polynomial regression smoothing of the profile plots, while the blue dots 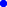 and vertical lines 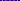 represent the minima found

To correlate periodic variation in the element concentration to the observed lens rings, the distance between each lens ring was used to parse each LA‐ICPMS measurement to the corresponding ring. By overlapping the regression lines with the annotated rings, it was then possible to look for coherence in maxima and minima, and the visible rings, as illustrated in Figure 3. To test whether there is a generic correlation in possible patterns, a mixed‐effects model between the distance from element core to local minima or maxima, and distance from lens core to visible rings were set up as follows, with fishID included as a random effect:

(2) $$Y_{i}=\mu +a_{1}\left(\text{lens}\_\text{Ring}\right)+d\left(\text{fishID}\right)+\varepsilon_{i}$$

where *Y* represents local minima or maxima in the given element profiles, *i = 1*, *… n* is the number of consecutive minima, *d*(fishID) ∼ *N*(0,*σ*_fishID_^2^) and $\varepsilon_{i}∼N\left(0,\sigma^{2}\right)$.

## 3 RESULTS

### 3.1 Element concentrations and trends in eye lenses

**FIGURE 4:**
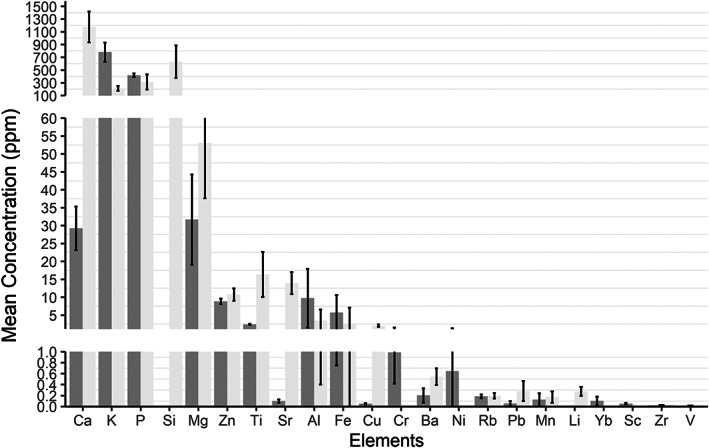
*Gadus morhua* eye lens element concentrations in ppm measured by solution ICP‐MS (dark grey, *n* = 3) and LA ICP‐MS (light grey, *n* = 12), respectively. Data shown are mean with standard error bars; the *y* axis is truncated at intermittent intervals for the best visual representation of differences in concentration between the two methods

For the dissolved eye lenses, measurable concentrations of 22 isotopes (^27^Al, ^137^Ba, ^43^Ca, ^52^Cr, ^65^Cu, ^57^Fe, ^39^K, ^7^Li, ^25^Mg, ^55^Mn, ^61^Ni, ^31^P, ^208^Pb, ^85^Rb, ^45^Sc, ^29^Si, ^88^Sr, ^47^Ti, ^50^V, ^149^Yb, ^66^Zn, ^90^Zr) were identified using solution ICP‐MS. LA‐ICPMS‐derived mean transect concentrations, obtained by averaging the values of the entire transect of the whole eye lenses, matched the solution ICP‐MS values as shown in Figure 4. A good match could be seen for many of the elements, but Ca, Sr and Cu varied greatly and a few elements were not represented for both analysis methods. The concentrations obtained by solution and laser ablation ICPMS do not originate from the same individuals. Thus, there may be interindividual variations, *e*.*g*., due to a less homogeneous occurrence of certain elements, that affect the results. Nevertheless, this comparison demonstrates that it is possible to quantify, with reasonably good accuracy and precision, most of the elements with abundances even below 1–2 ppm by LA‐ICPMS. Exceptions are Ca, Cu and Sr, which differ considerably between the two methods. For Cu and Sr this is attributable to the fact that the solution ICP‐MS instrumentation used in this study for some matrixes underestimates these elements severely. Improvements in the LA‐ICPMS methodology for measuring Ca is also required, in particular for spatial Ca variations occurring across the eye lenses. The analytical LA‐ICPMS approach used is thus suitable for analysing eye lenses with respect to most of the measured elements and their abundance range, except for Ca, whereas Cu and Sr are considered to be somewhat more applicable. Thus, irrespective of the difference in concentration for these elements between the two analytical methods, element ratios obtained by LA‐ICPMS should be usable for at least qualitative interpretations.

**FIGURE 5:**
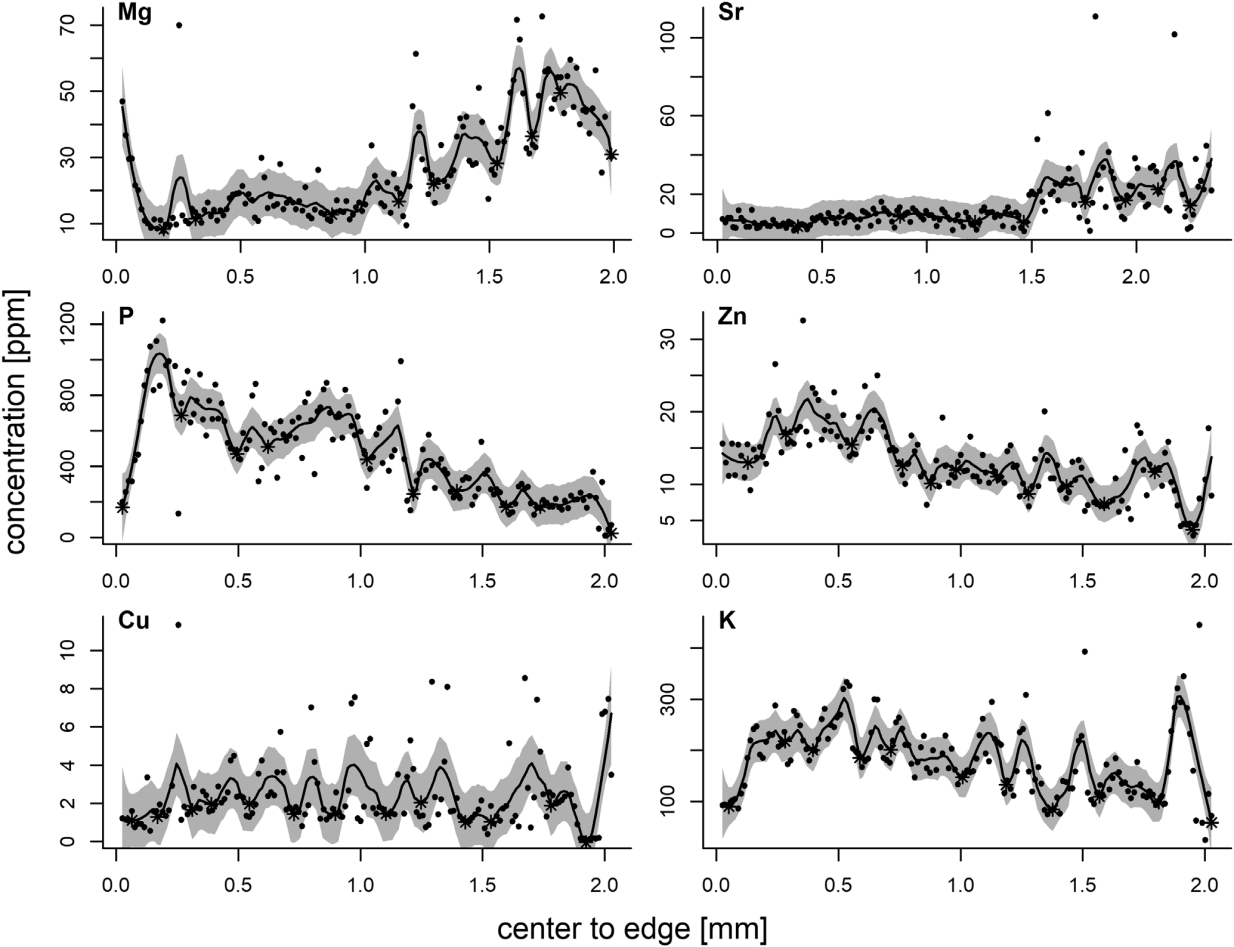
Example of *Gadus morhus* eye lens element concentration profiles of a typical individual for Mg, Sr, P, Zn, Cu and K. Profiles are shown from core to edge. Also shown is the local second‐degree polynomial regression and its confidence band, together with detected minima marked with a star

In general, element concentration profile plots across the eye lenses along the transect demonstrate coherent trends for almost all individuals. The alkaline earth metals Mg and Sr typically show an increasing abundance from core to edge of the eye lenses and Mg shows a fairly prominent peak domain from the core to edge area. The elements P and Zn show an opposite decreasing trend from the core region towards the edge, but P has similar‐looking peak trends prominent in the core area. The elements Cu and K only show decreasing abundances in a fairly narrow field near the core and edge of the eye lenses profiles and otherwise stable fluctuating element profiles across the transect of the eye lenses. Examples of these trends are shown in Figure 5.

**FIGURE 6:**
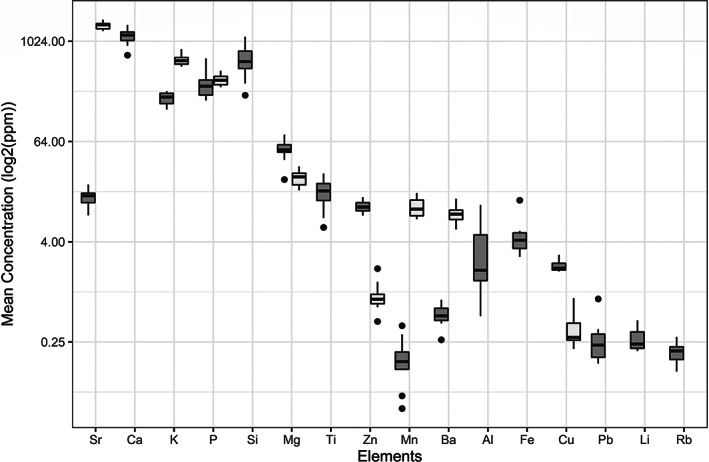
Box plot of the mean concentrations (*n* = 12) in ppm of elements in the *Gadus morhua* eye lenses (dark grey) and the otoliths (light grey) measured by LA ICP‐MS (*y* axis in log2 scale). Boxplot shows the mean (solid line 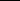), interquartile range (box 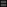), largest and smallest values within 1.5 times the interquartile range outside the box (whiskers 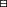) and outliers (dots 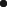)

Going forward with analysis and comparison of lens and otolith using LA‐IPCMS data, nine elements were included for the otolith analyses (Ba, Ca, Cu, K, Mg, Mn, P, Sr, Zn), with an additional seven elements included for the eye lenses (Al, Fe, Li, Pb, Rb, Si, Ti). Element concentrations, together with the standard deviation and SNR value, are shown in Figure 6 and Table 2.

**TABLE 2:** Overview of the chemical data available for the 12 Baltic cod (*Gadus morhua)*, where both otolith and eye lenses were analysed

	Otolith (ppm)			Eye lens (ppm)		
Element	Mean	S.D.	SNR	Mean	S.D.	SNR
^27^Al	–	–	–	3.47	3.07	1.28
^137^Ba	8.79	2.25	15.30	0.54	0.15	12.61
^43^Ca	3.63e5	1.01e4	1297.75	1177.11	241.32	23.79
^65^Cu	0.37	0.20	3.47	2.07	0.33	38.93
^57^Fe	–	–	–	2.41	4.70	0.26
^39^K	620.61	103.97	35.63	212.60	36.73	33.50
^7^Li	–	–	–	0.28	0.08	11.01
^25^Mg	23.50	4.74	24.58	53.08	15.46	11.78
^55^Mn	10.57	2.83	13.91	0.17	0.10	2.68
^31^P	354.99	49.13	52.21	314.18	119.80	6.88
^208^Pb	–	–	–	0.28	0.18	2.33
^85^Rb	–	–	–	0.20	0.051	14.50
^29^Si	–	–	–	631.21	255.64	6.10
^88^Sr	1582.62	164.1	93.00	13.94	3.07	20.63
^47^Ti	–	–	–	16.39	6.28	6.81
^66^Zn	0.91	0.38	5.79	10.74	1.70	39.83

*Note*: The values are from all the fishes grouped together. s.d., standard deviation; SNR, signal to noise ratio.

### 3.2 Comparison of eye lens and otolith chemical features

Of the elements included in this study, seven (Ba, Cu, K, Mg, P, Sr, Zn*)* had, when taking the mean over the whole growth axis, SNR > 5 for both the lens and otolith. These elements were modelled individually with model (1). No significance of any factors included was found for P. This implies that a general difference in concentration between lens and otolith could not be demonstrated. For all remaining elements the model could be reduced, excluding the factor *sex* and the interaction of *sex* and *type*. In the reduced model *type* was found to be significant for all remaining elements (ANOVA, num d.f. = 1, *P* < 0.05; for further details see Supporting Information Table S2), indicating a general difference between lens and otolith element concentrations, with Ba, K and Sr being significant lower in the lens than otolith and Cu, Mg and Zn being significantly higher.

For data representing the mean ‘core’ and ‘edge’, SNR > 5 for both lens and otolith occurs for the elements K, Mg and P in the ‘edge’ data and for Cu and Zn in the ‘core’ data. The relevant elements were analysed using model (1) for the ‘core’ data and model (2) for the ‘edge’ data. For all ‘core’ and ‘edge’ data, the model reduction resulted in exclusion of *sex* and its interactions. A significant influence of *type* was found on all elements modelled (ANOVA, d.f. = 1, *P* < 0.05; for further details see Supporting Information Table S2).

Through these analyses, life‐stage‐specific trends in P and Mg were discovered, highlighting that element concentrations averaged over the entire tissue mask ontogenetic changes between the two structures, where lens concentrations in P are higher than otolith concentrations in the core, but lower in the edge. Such ontogenetic patterns are also observed in Mg, but with a mirror‐like inverse pattern compared to P, both illustrated in Supporting Information Figure S3.

### 3.3 Comparison of eye lens ring and chemistry

**FIGURE 7:**
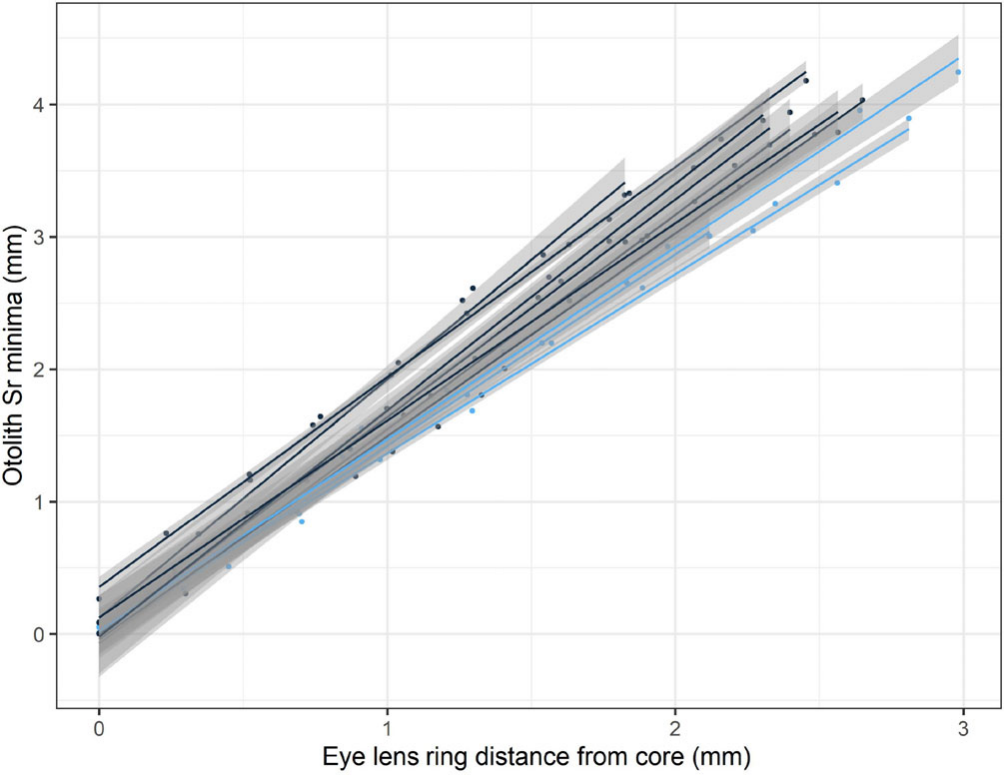
Linear regression of the distance of otolith Sr minima from the core (see Figure 5) against the corresponding eye lens rings distance from the core of *Gadus morhua*. The figure shows mean correlation with standard error for each individual separately. Blue, the individuals where the lens rings 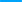 were considered easy to identify; Black, lens rings 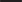 were difficult to identify (fish IDs #6 and #11 could not be plotted due to unequal *x* and *y* data)

The local second‐degree polynomial regression smoothing of the profile plots of the lens elements shows coherent profile trends across all individuals. When overlaying the element profile plots from the otoliths with the lens rings, a clear coherence was found between the maxima and minima of otolith Sr and the lens rings (Figure 3), where each minimum in otolith Sr coincides with a ring in the eye lens. This trend was observed for most of the individuals included herein, as shown on Figure 7, where the straight lines with small confidence bands of the linear regression illustrate the correlations between the lens rings and the otolith Sr minima found. IDs #6 and #11 could not be plotted since they had differing numbers of points; for these two individuals the lens rings were considered easy to identify. The results of model 2 confirm the correlation found, with the effect of lens rings being very significant (ANAOVA, sum sq = 143, mean sq = 143, num d.f. = 1, den d.f. = 84.5, *P* < 0.05). Contrary to this, all other elements from either the lenses or otoliths did not correlate with the observed lens rings, having maxima and minima not coinciding with ring patterns of the same individual.

## 4 DISCUSSION

At least 50 elements have been detected in otoliths using various methods (Campana, 1999; Hüssy *et al*., 2020; Sturrock *et al*., 2012). Likewise, several elements have previously also been detected in fish eye lenses, but these were never explored by LA‐ICPMS analysis. To our knowledge, our study is the first fish eye lens investigation using LA‐ICPMS. It thus serves as a baseline study for future investigations in respect to which elements can be anticipated to occur in fish eye lenses (at least from cod) and which elements may be too scarce or too noisy to be useful when using comparable analytical equipment. By comparing the two analytical methods applied here to the eye lens, it is apparent that for the heavier metals found only in trace amounts of <0.1 ppm (Yb, Sc, Zr, V), solution ICP‐MS analysis is preferable to LA‐ICPMS. On the other hand, the LA‐ICPMS technique not only provides a very high spatial resolution across the measured object, but also demonstrates reliable concentration determination for the elements Al, Ba, Fe, K, Li, Mg, Mn, P, Pb, Rb, Si, Sr, Ti and Zn, whereas results for Cr, Cu and Ca should be further investigated.

Other studies reporting trace element concentrations in fish eye lenses (Dove, 1999; Dove & Kingsford, 1998; Gillanders, 2001; Kingsford & Gillanders, 2000; Pourang *et al*., 2018; Quaeck, 2017; Quaeck‐Davies *et al*., 2018) used dissolution procedures, thereby missing the spatial resolution. The concentrations reported in these studies largely correspond to the concentrations from the solution‐based analysis reported here, or are at least on the same order of magnitude. The major difference is that our study reports the entire suite of elements that were found in eye lenses, while earlier studies seem restricted to a few elements. When comparing the relative offset of eye lens concentrations to the otolith concentration of the elements Mn, Ba and Pb in Gillanders (2001) to the LA‐ICPMS results from this study, they appear to follow the same trends relative to the otolith concentration, whereas Sr clearly differs, having a higher relative concentration to the otolith. Variation in concentrations between studies are to be expected since interspecies differences and differences in environment are bound to have a major impact.

In general, there are large elemental differences between eye lenses and otoliths, and for the most part there is no real correlation in mean concentrations. This is, however, not surprising, as otoliths are composed of calcium carbonate and eye lenses consist of a variety of proteins (Wistow & Slingsby, 2016). While our analyses on a relatively small sample indicated that sex was not a significant driver of the observed trends, it would still be useful to include more physiological features, such as maturity stage, age, somatic condition *etc*., to identify where the interindividual variation in element concentrations comes from. However, this requires a targeted sampling design with a much larger sample size.

An opposing pattern was observed between eye lens P and Mg concentrations, with high P and low Mg near the core, and a steadily decrease (P) and increase (Mg) towards the edge of the eye lenses. This trend seems to be proportional, but further investigation of the detailed behaviour of this feature is required. A quantitative analysis of this relationship was not possible here since P and Mg exhibit an inverse relationship that is associated with an instrumental/data reduction artefact when calculating the total concentrations. Another interesting trend regarding P and Mg is demonstrated in the relationship between eye lens and otolith tissues. For a given section of the data transect, when P is significantly higher in the eye lens than in the otolith then Mg is consistently higher in the otolith. This relationship, which seems proportional at all points in the data transect, indicates a complex and interdependent relationship between lenses and otoliths, which suggests that the incorporation of P and Mg is largely regulated by biochemical and physiological processes.

When testing for coherence between the lens growth rings and patterns in otolith element concentration, minima in otolith Sr were found to correspond significantly with the lens rings for all but two of the individuals. In these the number of maxima and rings differed, with one and wo more rings than minima present, although overlap between other rings and minima were still present. It should be considered here that the model which showed strong correlation between lens rings distances and otolith Sr valley distances also did not include the two individuals previously mentioned. The exclusion of theses two individuals thus questions whether this is a general trend or only true for some individuals, and emphasizes the importance of quality control of eye lens ring identification. In otoliths, Sr concentration is known to have a positive correlation with the extrinsic environmental concentration, where environmental Sr concentration typically explains >90% of otolith Sr concentration and other variables like salinity and temperature only have a limited effect (Hüssy *et al*., 2020 and references therein). Cod in the Baltic Sea experience large seasonal variations in ambient salinity and oxygen concentrations as a result of horizontal and vertical migrations related to spawning and feeding, respectively. During spawning, the eastern Baltic cod moves to deeper and more saline waters, while the feeding areas are predominantly located in shallower areas where the water is less saline (Righton *et al*., 2010; Schaber *et al*., 2012). The present results do not provide evidence of the mechanisms driving the observed correspondence in otolith element and eye lens ring patterns. However, the close match suggests a link between seasonal migrations associated with variations in environmental Sr and eye lens ring formation.

Some notable observations can be made between how easy it is to discern the eye lens rings in relation to the element profile plots. When P and Mg have continuous opposing downward and upward trends, respectively, without major peaks, the lens rings are better defined. We thereby speculate whether these profile trends are connected to changes in the crystalline structure of the eye lens, from alpha to gamma as the fish ages (Dove, 1999). Additionally, the otolith Sr maxima matched the lens growth rings best in the individuals where the rings were less clear. It is not within the scope of this study to try to infer the physiological mechanisms or influence of environmental factors behind these results. Nevertheless, these features certainly require further in‐depth investigations to identify the underlying mechanism behind the correlations.

The present study was based on only 12 individuals with unequal distribution of sex, capture date, *etc*. The sampling strategy has possible implications for the results in that it entrains an unbalanced design where model results are less robust than for larger studies. In addition, the standardization procedures and analytical approach might also be improved, here to use other elements as internal standards, such as K or Si, because these elements are an abundant and omnipresent constituent in eye lenses, further allowing for better analysis on the covariance of P and Mg. An additional challenge, presumably introducing some variability into the interpretation of the eye lens rings, is that no prior knowledge on how to identify the proper rings is currently available. It appears that the lens rings do not follow a consistent pattern, being incrementally closer to one another, as is observed for otolith annuli. The rings near the edge appear closer to one another, but they are very difficult to distinguish from one another and should be subject to further examination.

## 5 CONCLUSIONS AND FUTURE CONSIDERATIONS

This study demonstrates that LA‐ICPMS analyses can be conducted *in situ* on eye lenses with an acceptably high precision and accuracy using the analytical approach and instrumentation employed herein. Robust quantitative analyses have been produced providing reliable results, and thus the technique is shown to be a powerful tool for the interpretation of environmental, ecological and whole life history of fish.

Furthermore, the results of this study suggest that incorporation of trace elements is mostly governed by different processes for otoliths and eye lenses. The rings observed in cod eye lenses do not represent seasonal fluctuations or other age‐related measures directly, and the lens element patterns do not seem to correspond to these rings. However, an important result of this study is that patterns in otolith Sr generally match well with eye lens growth rings. Eye lenses may thus provide ecological and biological information on a fish's life that could complement otolith‐derived information.

The physiological mechanisms behind our results are still not fully established, but point towards a potential link between eye lens growth ring formation and fish behaviour or other regularly recurring drivers. It is, for instance, unclear from this study whether the observed patterns in Mg and P could be a derived effect from α/β crystalline formation or exchange, where protein structures reach a given length before their growth is terminated and a new growth layer starts to form.

It must be noted that the number of fish individuals used for this study does not allow major conclusions to be drawn on a larger scale and caution should be used in respect to other fish species. Further investigations are required to constrain or confirm our results. However, it does provide some important first results towards a better understanding of the use of fish eye lenses as a tool to unravel ecological and biological questions.

One weakness of our study was the sampling design, where a more balanced sampling design collecting a larger sample size and consideration for equal numbers of sex and maturity stage should be followed in future investigations. A greater contrast in the ages and lengths of individuals is also advisable, including quality assurance of a consistent interpretation of the eye lens growth rings.

Future studies should therefore (a) identify the physiological processes regulating the formation of the growth rings occurring in eye lenses as well as the temporal aspects of their formation periodicity through targeted laboratory and field experiments; (b) rigorously test incorporation mechanisms of the different elements into the growing lens structure; and (c) improve on the standardization and analytical approach aiming at establishing a generally accepted analytical protocol.

## AUTHOR CONTRIBUTIONS

The work is original and all authors approved the submitted manuscript. K.H. conceived the study design, K.H. collected the samples, K.H., T.B.T. and B.D.H. processed and analysed the samples, J.S analysed the data and J.S. wrote the manuscript. All authors critically reviewed the manuscript and gave comments.

## FUNDING INFORMATION

This study was funded by the Danish Ministry for Environment and Food, and the European Maritime Fisheries Fund (grant No. 33113‐B‐19‐140).

## Supporting information


**Supporting Information Figure S1** Accuracy and precision of LA ICPMS data. Measured laser concentrations (ppm) compared to reference values (ppm) for the five standards used (see Supporting Information Table S1). The internal measurement precision for line transect analyses of all standards were less than 10% (2SE) except for P in NIST‐614 and Cu in FEBS‐1, which were less than 20% (2SE). Generally, the accuracy of measured elements was within 10% and no more than 1 ppm. For FEBS‐1, elements with less than 50 ppm were measured to have higher concentrations. Ba, Cu and Zn with reference values of 5–6 ppm were measured to be 10–20 ppm. Mn with a reference value of 0.69 was measured to be 10–11 ppm. Mg with a reference value of 24 ppm was measured to be 300–700 ppm. Sr measurements were both precise and accurate for all standards. This figure is equivalent to Fig. S5 in Albertsen *et al*. (2021) in the present paper is based on the same data as Albertsen *et al*.

**Supporting Information Figure S2** Example of *Gadus morhua* eye lens *Mg* concentration along a transect from edge to edge of a Baltic cod (sex = female, length = 43.4 cm, caught in January 2017). The vertical lines illustrate the partitioning of data for outlier detection, where the red points illustrate outliers that were removed as they were larger or smaller than *μ* ± 4*σ*

**Supporting Information Figure S3** Boxplot of P and Mg concentrations from *Gadus morhua* eye lenses (dark grey *n* = 12) and otoliths (light grey *n* = 12), respectively, representing overall mean as well as core and edge concentrations. Stars indicate significant differences between structures. The boxplot shows the mean (solid line), interquartile range (box), and largest and smallest values within the 1.5 times interquartile range outside the box (whiskers) and outliers (dots)

**Supporting Information Table S1** LA‐ICP‐MS analytical setup at the Geological Survey of Denmark and Greenland

**Supporting Information Table S2** ANOVA results of the models run for various elements
