# Supplementary figures and images for: Eyes and ears: A comparative approach linking the chemical composition of cod otoliths and eye lenses
Source: J Fish Biol. 2022 Jul 29;101(4):985–95. doi: 10.1111/jfb.15159 (PMC9796464; doi:10.1111/jfb.15159)

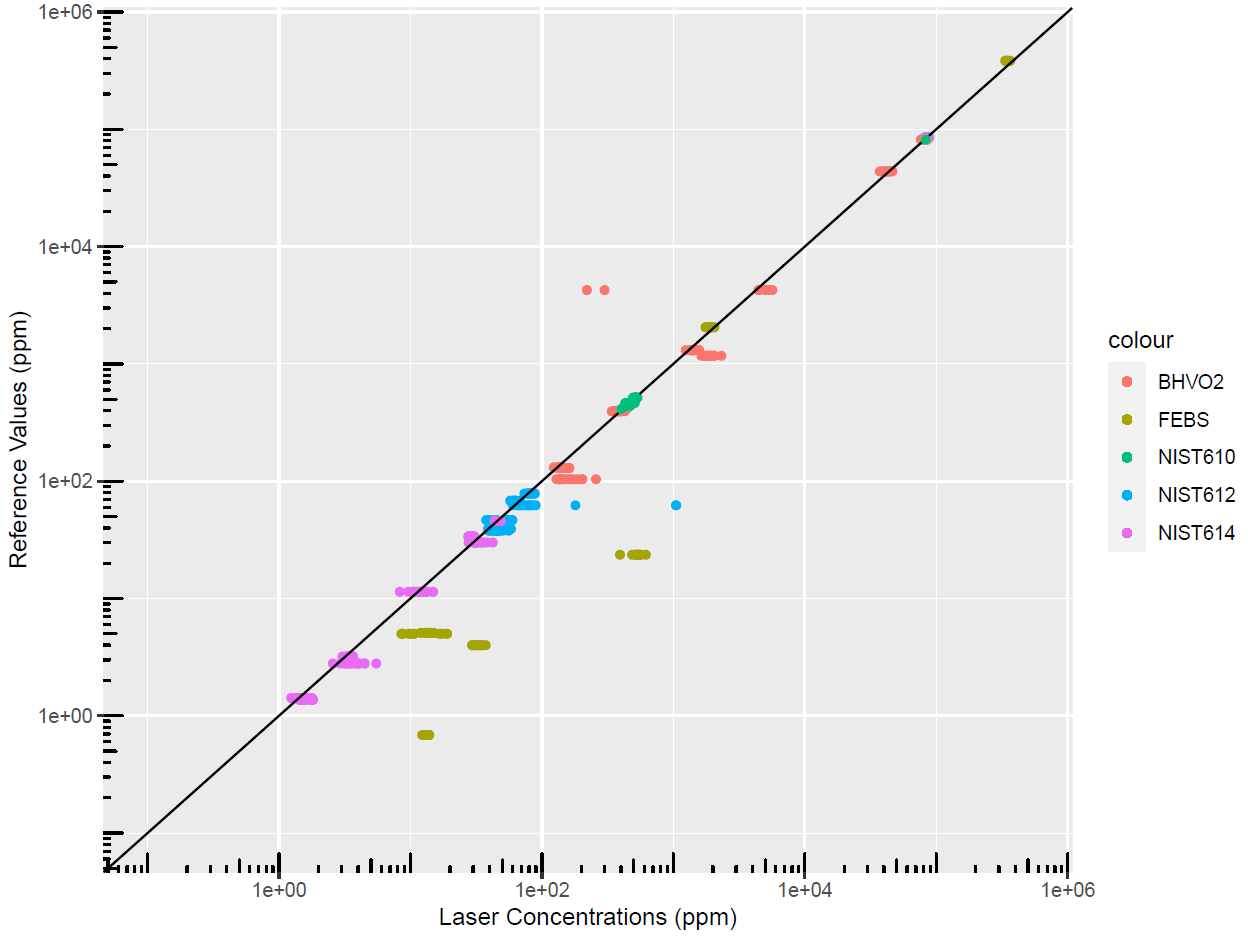

Supplement: Supplementary file 1 — Supporting Information Figure S1 Accuracy and precision of LA ICPMS data. Measured laser concentrations (ppm) compared to reference values (ppm) for the five standards used (see Supporting Information Table S1). The internal measurement precision for line transect analyses of all standards were less than 10% (2SE) except for P in NIST‐614 and Cu in FEBS‐1, which were less than 20% (2SE). Generally, the accuracy of measured elements was within 10% and no more than 1 ppm. For FEBS‐1, elements with less than 50 ppm were measured to have higher concentrations. Ba, Cu and Zn with reference values of 5–6 ppm were measured to be 10–20 ppm. Mn with a reference value of 0.69 was measured to be 10–11 ppm. Mg with a reference value of 24 ppm was measured to be 300–700 ppm. Sr measurements were both precise and accurate for all standards. This figure is equivalent to Fig. S5 in Albertsen et al. (2021) in the present paper is based on the same data as Albertsen et al. [file JFB-101-985-s001.png]

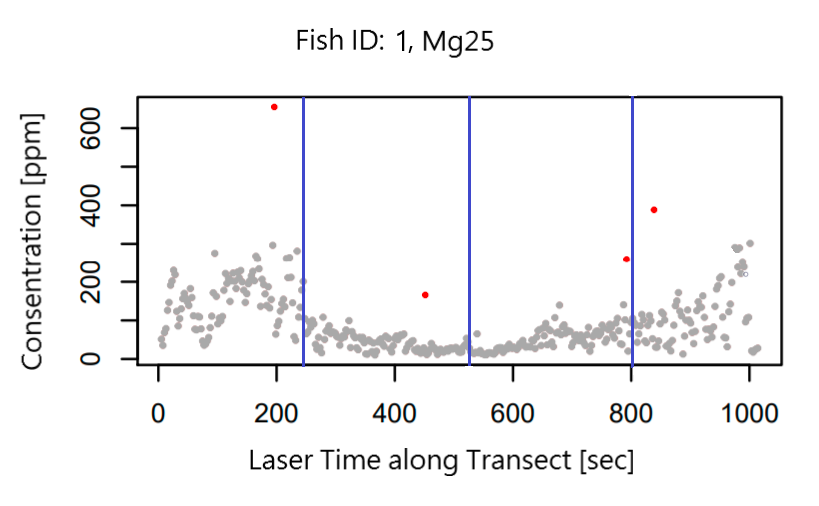

Supplement: Supplementary file 2 — Supporting Information Figure S2 Example of Gadus morhua eye lens Mg concentration along a transect from edge to edge of a Baltic cod (sex = female, length = 43.4 cm, caught in January 2017). The vertical lines illustrate the partitioning of data for outlier detection, where the red points illustrate outliers that were removed as they were larger or smaller than μ ± 4σ [file JFB-101-985-s002.png]

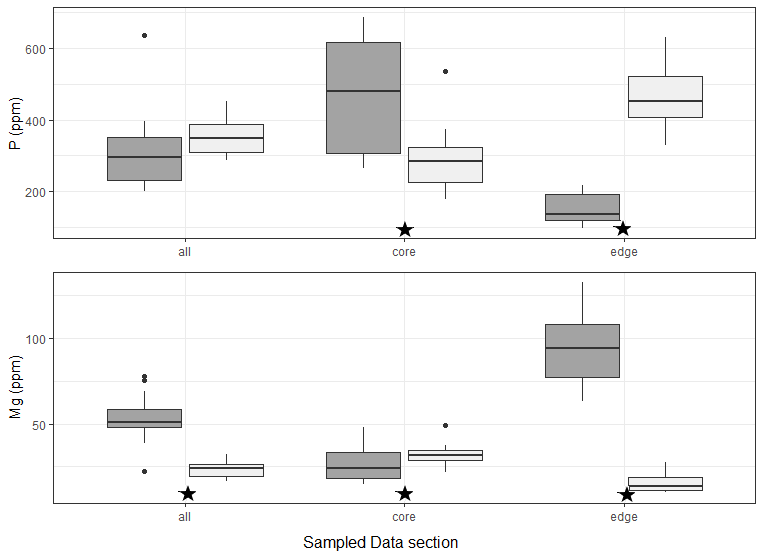

Supplement: Supplementary file 3 — Supporting Information Figure S3 Boxplot of P and Mg concentrations from Gadus morhua eye lenses (dark grey n = 12) and otoliths (light grey n = 12), respectively, representing overall mean as well as core and edge concentrations. Stars indicate significant differences between structures. The boxplot shows the mean (solid line), interquartile range (box), and largest and smallest values within the 1.5 times interquartile range outside the box (whiskers) and outliers (dots) [file JFB-101-985-s005.png]
