# Supplementary material for: Eyes and ears: A comparative approach linking the chemical composition of cod otoliths and eye lenses
Source: J Fish Biol. 2022 Jul 29;101(4):985–95. doi: 10.1111/jfb.15159 (PMC9796464; doi:10.1111/jfb.15159)
Supplement: Supplementary file 4 — Supporting Information Table S1 LA‐ICP‐MS analytical setup at the Geological Survey of Denmark and Greenland [file JFB-101-985-s004.docx]

**Table S1.** LA- ICP-MS analytical setup at the Geological Survey of Denmark and Greenland.

| **Laser ablation system** |  |
| --- | --- |
| Instrumentation | NWR 213 solid state laser (Elemental Scientific Lasers) |
| Ablation cell type | Standard Vol. 2 cell |
| Laser wavelength | 213 nm |
| Pulse width | ~4 ns |
| Fluence | 8-9 J/cm2 |
| Repetition rate | 10 Hz |
| Scan speed | 5 µm/s |
| Spot size | 40 µm circular |
| Background collection | 30 seconds (and 30 s washout after analysis) |
| Single analysis duration | 2-20 minutes per otolith/eye lens (line scan) |
| Cell carrier gas flow | ~850-920 ml/min He |
| **ICP-MS Instrument** |  |
| Instrumentation | Thermo-Fisher Scientific Element2 Magnetic Sector Field HR-ICP-MS |
| Mass resolution | 300 (low) |
| Cones | Ni |
| Forward power (RF) | 1450 W |
| Plasma gas flow | 16 L/min Ar |
| Auxiliary gas flow | 0.90 L/min Ar |
| Oxide production rate | Tuned to ≤ 0.3% UO_2_ (^238^U^16^O_2_/^238^U) |
| Masses measured | ***Otoliths***: ^25^Mg, ^31^P, ^43^Ca, ^44^Ca, ^55^Mn, ^63^Cu, ^64^Zn, ^85^Rb, ^88^Sr, ^137^Ba  ***Eye lenses***: ^7^Li, ^25^Mg, ^27^Al, ^29^Si, ^31^P, ^39^K, ^43^Ca, ^45^Sc, ^47^Ti, ^50^V, ^52^Cr, ^55^Mn, ^57^Fe, ^65^Cu, ^66^Zn, ^85^Rb, ^88^Sr, ^90^Zr, ^137^Ba, ^149^Yb, ^208^Pb |
| Dwell time (ms) | 10 ms |
| Samples per peak | 10 |
| **Data Processing** |  |
| Software | Iolite v. 2.5 using the Trace_Elements_IS DRS (Paton et al. 2011; Hellstrom et al. (2008) |
| Primary standard | NIST-610 glass (preferred values from Jochum *et al.* 2011) |
| Secondary standard(s) | NIST612 (glass tablet; preferred values from Jochum et al. 2011), NIST-614 (glass tablet; preferred values from Jochum et al. 2011), FEBS-1 otolith (*Lutjanus campechanus*; NRCC data) and BHVO-2 (basalt powder; preferred values from Jochum et al. 2016) |
| Internal standard isotope | ^43^Ca (Ca concentration set to 38.3 wt% Ca for all otoliths)  ^31^P (P concentration set to 0.09 wt.% P (393 ppm) for all eye lenses) |
