# Supplementary material for: Eyes and ears: A comparative approach linking the chemical composition of cod otoliths and eye lenses
Source: J Fish Biol. 2022 Jul 29;101(4):985–95. doi: 10.1111/jfb.15159 (PMC9796464; doi:10.1111/jfb.15159)
Supplement: Supplementary file 5 — Supporting Information Table S2 ANOVA results of the models run for various elements [file JFB-101-985-s003.docx]

| Data Type | Element | Transf. | Effect | Mean Sq | Num df | Den df | Pr(>F) |
| --- | --- | --- | --- | --- | --- | --- | --- |
| ”all” | *Ba* | Log() | *type* | 46.793 | 1 | 22 | < 0.05 |
| ”all” | *Cu* | - | *type* | 17.188 | 1 | 22 | < 0.05 |
| ”all” | *K* | ^0.5 | *type* | 637.52 | 1 | 22 | < 0.05 |
| ”all” | *Mg* | Log() | *type* | 3.7314 | 1 | 22 | < 0.05 |
| ”all” | *P* | - | *type* | 9994.9 | 1 | 11 | 0.29 |
| ”all” | *Sr* | Log() | *type* | 135.39 | 1 | 11 | < 0.05 |
| ”all” | *Zn* | - | *type* | 32.583 | 1 | 11 | < 0.05 |
|  |  |  |  |  |  |  |  |
| ”edge” | *K* | Log() | *type* | 10.961 | 1 | 22 | < 0.05 |
| ”edge” | *Mg* | ^0.5 | *type* | 197.37 | 1 | 11 | < 0.05 |
| ”edge” | *P* | - | *type* | 606155 | 1 | 22 | < 0.05 |
|  |  |  |  |  |  |  |  |
| ”core” | *Cu* | Log() | *type* | 8.4238 | 1 | 11 | < 0.05 |
| ”core” | *K* | ^0.5 | *type* | 1431.1 | 1 | 22 | < 0.05 |
| ”core” | *Mg* | Log() | *type* | 0.3929 | 1 | 11 | < 0.05 |
| ”core” | *P* | - | *type* | 191866 | 1 | 22 | < 0.05 |
| ”core” | *Zn* | ^0.5 | *type* | 39.119 | 1 | 22 | < 0.05 |

**Table S2:** Results of ANOVA tests, for the reduced model 1 ($Y_{i}= \mu+a_{1}\left( type_{i} \right)+d\left( fishID \right)+ \varepsilon_{i}$), for elements with SNR > 5 for the whole data transect, the core and the edge data respectively. The model input for all instances are 24 observations in groups: *fishID*, 12 observations. Transf. = model transformation, Effect = effect tested, Mean Sq = Mean square, Num df = numerator degrees of freedom, Den df = denominator degrees of freedom, Pr(>F) = p value.
